# The mechanism for the radioprotective effects of zymosan‐A in mice

**DOI:** 10.1111/jcmm.13538

**Published:** 2018-02-07

**Authors:** Jicong Du, Pei Zhang, Hainan Zhao, Suhe Dong, Yanyong Yang, Jianguo Cui, Fu Gao, Jianming Cai, Cong Liu

**Affiliations:** ^1^ Department of Radiation Medicine Faculty of Naval Medicine Second Military Medical University Shanghai China

**Keywords:** DNA damage, G‐CSF, GM‐CSF, radioprotection, RNA sequencing, zymosan‐A

## Abstract

It proved that Zymosan‐A protected the haematopoietic system from radiation‐induced damage via Toll‐Like Receptor2 in our previous study. In this study, we investigated the potential mechanism for the radioprotective effects of Zymosan‐A. The mice were treated with Zymosan‐A (50 mg/kg, dissolved in NS) via peritoneal injection 24 and 2 hours before ionizing radiation. Apoptosis of bone marrow cells and the levels of IL‐6, IL‐12, G‐CSF and GM‐CSF were evaluated by flow cytometry assay. DNA damage was determined by γ‐H2AX foci assay. In addition, RNA sequencing was performed to identify differentially expressed genes (DEGs). Zymosan‐A protected bone marrow cells from radiation‐induced apoptosis, up‐regulated IL‐6, IL‐12, G‐CSF and GM‐CSF in bone marrow cells. Zymosan‐A also protected cells from radiation‐induced DNA damage. Moreover, RNA sequencing analysis revealed that Zymosan‐A induced 131 DEGs involved in the regulation of immune system process and inflammatory response. The DEGs were mainly clustered in 18 KEGG pathways which were also associated with immune system processes. Zymosan‐A protected bone marrow cells from radiation‐induced apoptosis and up‐regulated IL‐6, IL‐12, G‐CSF and GM‐CSF. Moreover, Zymosan‐A might also exhibit radioprotective effects through regulating immune system process and inflammatory response. These results provided new knowledge regarding the radioprotective effect of Zymosan‐A.

## 1 INTRODUCTION

Acute radiation exposure often leads to serve damages to radiosensitive tissues, which limit the use of radiotherapy in clinic.^1^, ^2^, ^3^ Haematopoietic system is vulnerable to ionizing radiation (IR), and the failure of haematopoietic system is the major reason of death after acute radiation exposure.^4^, ^5^ Radiation‐induced injury is a complex pathophysiological process.^6^, ^7^ In general, DNA is the critical target of ionizing radiation. DNA damage caused by IR mediates inflammasome activation and cell death.^8^, ^9^, ^10^

Toll‐like receptors (TLRs) play essential roles in recognizing specific components of pathogenic microorganisms and triggering immune system responses.^11^, ^12^, ^13^, ^14^ In recent years, TLRs bring a new development direction to radioprotection. TLR2, TLR4, TLR5 and TLR9 have critical roles in radio‐resistance.^4^, ^15^, ^16^, ^17^, ^18^ In previous study, we showed that Zymosan‐A protected mice from radiation‐induced death, promoted cell viability and inhibited cell apoptosis caused by radiation *in vitro*. Using knockout mice, we proved that the radioprotective effects of Zymosan‐A were dependent on the TLR2 signalling pathway.^19^ In addition, we found that Zymosan‐A mitigated the damage of haematopoietic system and accelerated the recovery of haematopoiesis in mice. However, the potential mechanism is still unclear.

In this study, the molecular mechanism of radioprotection of Zymosan‐A was studied using flow cytometry, γ‐H2AX foci assay and RNA‐seq. Our experiments provided new knowledge regarding the radioprotective effect of Zymosan‐A.

## 2 MATERIALS AND METHODS

### 2.1 Chemicals and reagents

Zymosan‐A was purchased from Sigma–Aldrich Corp (St. Louis, MO, USA), and normal saline (NS) was obtained from ChangHai Hospital (Shanghai, China). The apoptosis detection kit was purchased from Invitrogen (Carlsbad, CA, USA). Anti‐Mouse GM‐CSF‐PE, Anti‐Mouse G‐CSF‐eFluor 660, Anti‐Mouse IL‐6‐PerCP‐eFluor 710, Anti‐Mouse IL‐12‐PE were purchased from BD‐Pharmingen (San Diego, CA, USA).

### 2.2 Cell culture and treatment

Human B lymphocyte (AHH‐1)) was obtained from American Type Culture Collection, and cultured in RPMI 1640 with 10% FBS at 37°C in a 5% CO2 humidified chamber. Cells were treated with Zymosan‐A (40 μg/mL) 12 and 2 hours before irradiation.

### 2.3 Animals and treatment

Male wild‐type C57BL/6 mice aged 6‐8 weeks were obtained from Chinese Academy of Sciences (Shanghai, China). All mice were housed in a laboratory animal room under standard conditions. The experiments were approved by the Laboratory Animal Center of the Second Military Medical University, China in conformance with the National Institute of Health Guide for the Care and Use of Laboratory Animals. The mice were treated with Zymosan‐A (50 mg/kg, dissolved in NS) via peritoneal injection 24 and 2 hours before ionizing radiation. Mice were sacrificed by cervical dislocation at different time after radiation.

### 2.4 Irradiation

^60^Co source in the radiation centre (Faculty of Naval Medicine, Second Military Medical University, China) was used to irradiate mice and cells. Mice were irradiated at 7.5 Gy, and cells were irradiated at 8.0 Gy at the rate of 1 Gy/min.

### 2.5 Antibody staining and flow cytometry

Bone marrow cells (BMCs) were isolated freshly. Then, cells were strained through a 40‐μm strainer in the presence of phosphate‐buffered saline and red blood cells were removed. Cells were stained with antibody for 20 minutes at 4°C. The cell apoptosis was analysed using the apoptosis detection kit according to the manufacturer's instructions. PI and Annexin V were used to stain BMCs. BMCs were fixed, permeabilized and labelled with anti‐G‐CSF, anti‐GM‐CSF, anti‐IL‐6 and anti‐IL‐12 and then subjected to flow cytometry analysis.

### 2.6 Immunofluorescence analysis

Immunofluorescence analysis was used to detect γ‐H2AX foci. AHH‐1 cells were seeded in 6‐well plates at the concentration of 2*10^5^ per well. Then, cells were treated with Zymosan‐A (40 μg/mL) 12 and 2 hours before 6 Gy irradiation. Then 0, 0.5, 2 hours later, cells were fixed in 4% paraformaldehyde for 20 minutes and permeabilized in 0.5% Triton X‐100 for 10 minutes. After blocked in BSA, cells were stained with γ‐H2AX, and then stained with the secondary antibody (1:1000). The images of cell smears were obtained using an Olympus BX60 fluorescent microscope (Olympus America Inc., Center Valley, PA, USA) equipped with a Retiga 2000R digital camera (Q Imaging Inc., Surrey, BC, Canada).

### 2.7 RNA sequencing and functional enrichment analysis

Total RNA was isolated from BMCs using Trizol (Invitrogen, USA) 24 hours after radiation. NanoVue (GE, USA) was used to assess RNA purity. Each RNA sample had an A260:A280 ratio greater than 1.8 and an A260:A230 ratio >2.0. RNA integrity was assessed using the Agilent 2200 Tape Station (Agilent Technologies, USA), and each sample had an RIN above 7.0. Briefly, mRNAs were isolated from the total RNA and fragmented to approximately 200 bp. Next, the collected mRNAs were subjected to first strand and second strand cDNA synthesis followed by adaptor ligation and enrichment with a low cycle according to the instructions provided with the TruSeq RNA LT/HT Sample Prep Kit (Illumina, USA). The purified library products were evaluated using the Agilent 2200 Tape Station and Qubit 2.0 (Life Technologies, USA). Sequencing was performed at Guangzhou Ribo Bio Co., Ltd. with the Illumina HiSeq 2500. Prior to sequencing, the raw data were filtered to produce high‐quality clean data. All the subsequent analyses were performed with the clean data. All the differentially expressed genes (DEGs) were used for heat map analysis, Gene Ontology Analysis and KEGG ontology enrichment analyses. For KEGG enrichment analysis, a *P*‐value < .05 was used as the threshold to determine significant enrichment of the gene sets.

### 2.8 Statistical analysis

Data were expressed as means ± standard deviation (SD). Two‐tailed Student's *t* test was used to analyse the difference between 2 groups. These data were analysed using SPSS ver. 19 (IBM Corp., Armonk, NY, USA). *P* < .05 was considered statistically significant.

## 3 RESULTS

### 3.1 Zymosan‐A inhibited BMCs apoptosis caused by radiation

**Figure 1:**
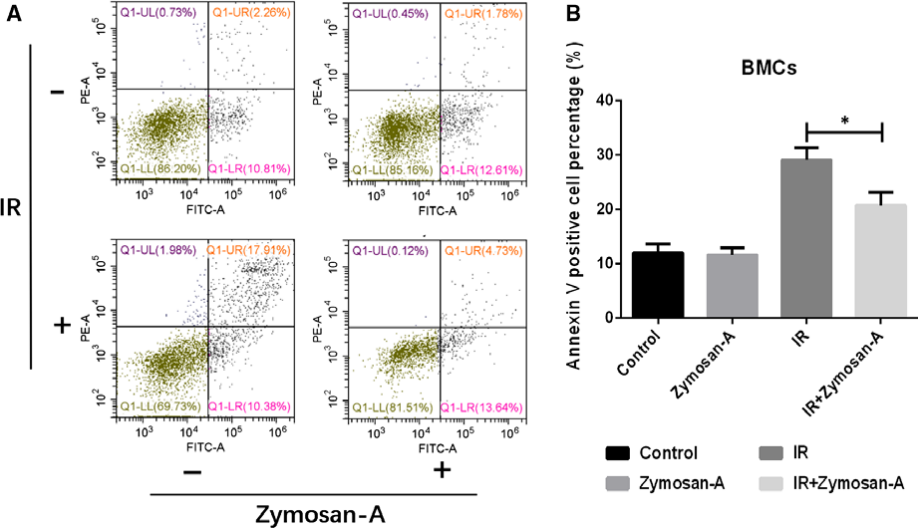
Zymosan‐A inhibited bone marrow cells (BMCs) apoptosis. (A) BMCs were isolated from mice 24 h after 7.5 Gy total body irradiation, and then the apoptosis of BMCs was analysed by flow cytometry. (B) Data are presented as mean ± SD of 3 independent experiments.

The mortality of mice after radiation was associated with a serious and continuous BMCs loss.^16^ In previous study, we showed that Zymosan‐A significantly improved the number of BMCs after ionizing radiation.^19^ To explore the potential mechanism, we detected the apoptosis of BMCs 24 hours after radiation. The results showed that the BMCs apoptosis rate increased after radiation, while the apoptosis rate was decreased significantly in BMCs from mice which treated with Zymosan‐A (Figure 1).

### 3.2 Zymosan‐A up‐regulated the levels of GM‐CSF, G‐CSF, IL‐12 and IL‐6 in BMCs

**Figure 2:**
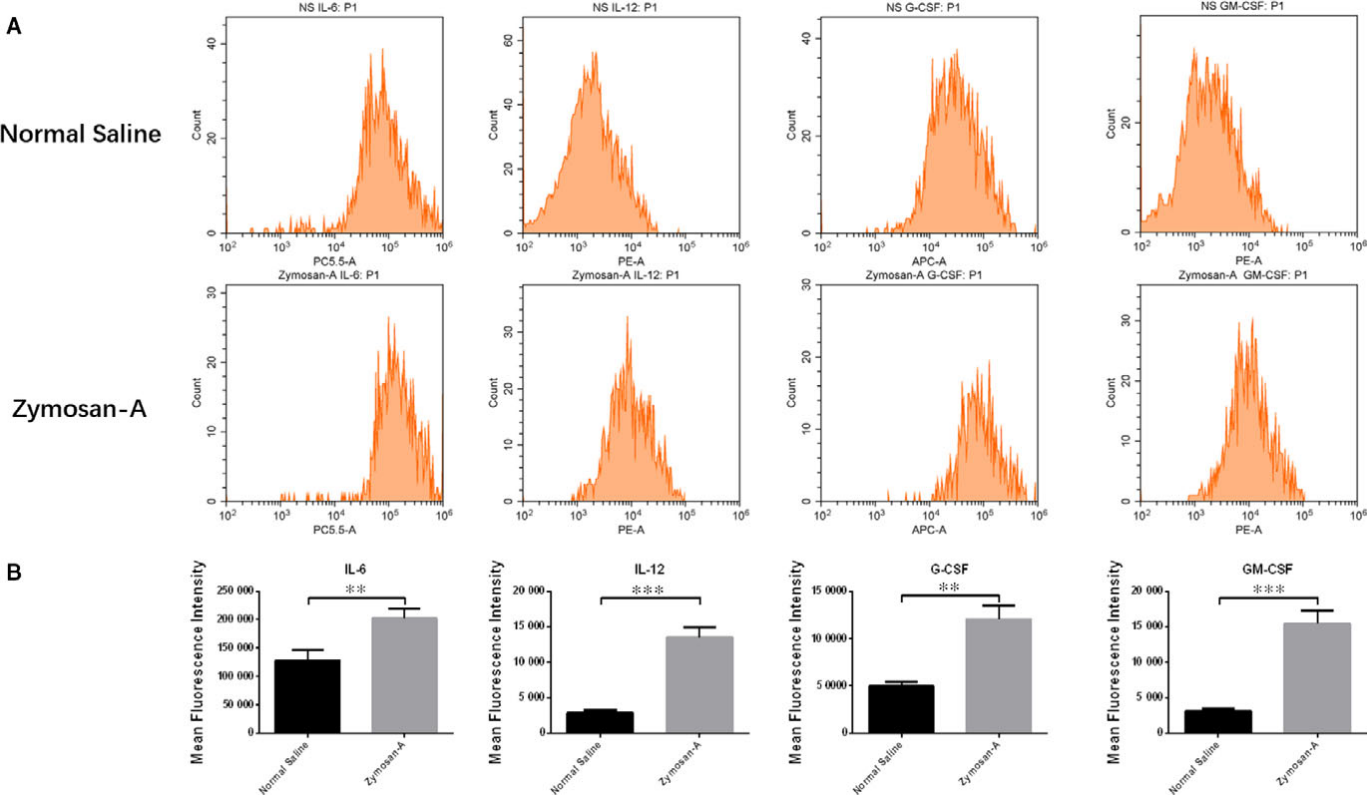
Zymosan‐A up‐regulated the level of GM‐CSF, G‐CSF, IL‐12 and IL‐6 in bone marrow cells (BMCs). BMCs were isolated from mice 24 h after radiation, and then the cytokines of BMCs were analysed by flow cytometry. (B) Data are presented as mean ± SD of 3 independent experiments.

The protective effects of GM‐CSF, G‐CSF, IL‐12 and IL‐6 have been proven in several studies.^20^, ^21^, ^22^ Those cytokines play important roles in the haematopoietic system.^23^, ^24^ Using flow cytometry, we found that Zymosan‐A up‐regulated the levels of GM‐CSF, G‐CSF, IL‐12 and IL‐6 in BMCs (Figure 2).

### 3.3 Zymosan‐A protected cells from radiation‐induced DNA damage

**Figure 3:**
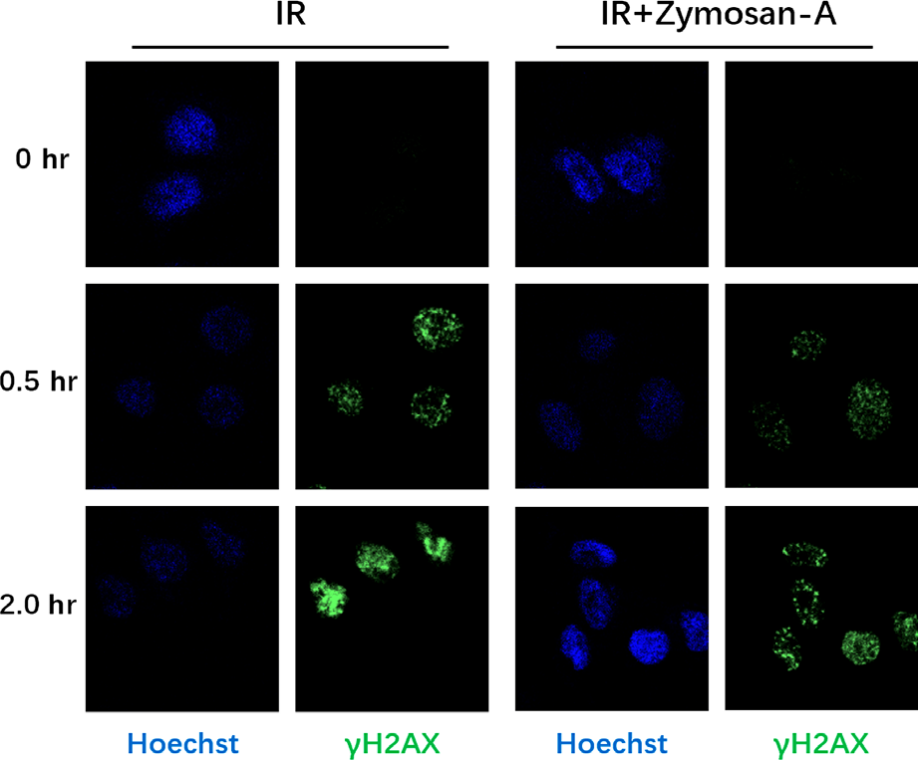
Zymosan‐A protected cells from radiation‐induced DNA damage. AHH‐1 Cells were irradiated at 8.0 Gy, and then the γH2AX foci were analysed.

DNA is a critical target of ionizing radiation. DNA damage caused by IR mediates inflammasome activation and cell death.^9^ The γ‐H2AX foci analysis was used to detect the effect on DNA repair of Zymosan‐A. This result demonstrated that Zymosan‐A reduced the number of γ‐H2AX foci per cell at 0, 0.5 and 2 hours after irradiation (Figure 3).

### 3.4 Identification of DEGs between IR + NS and IR + Zymosan‐A groups

**Figure 4:**
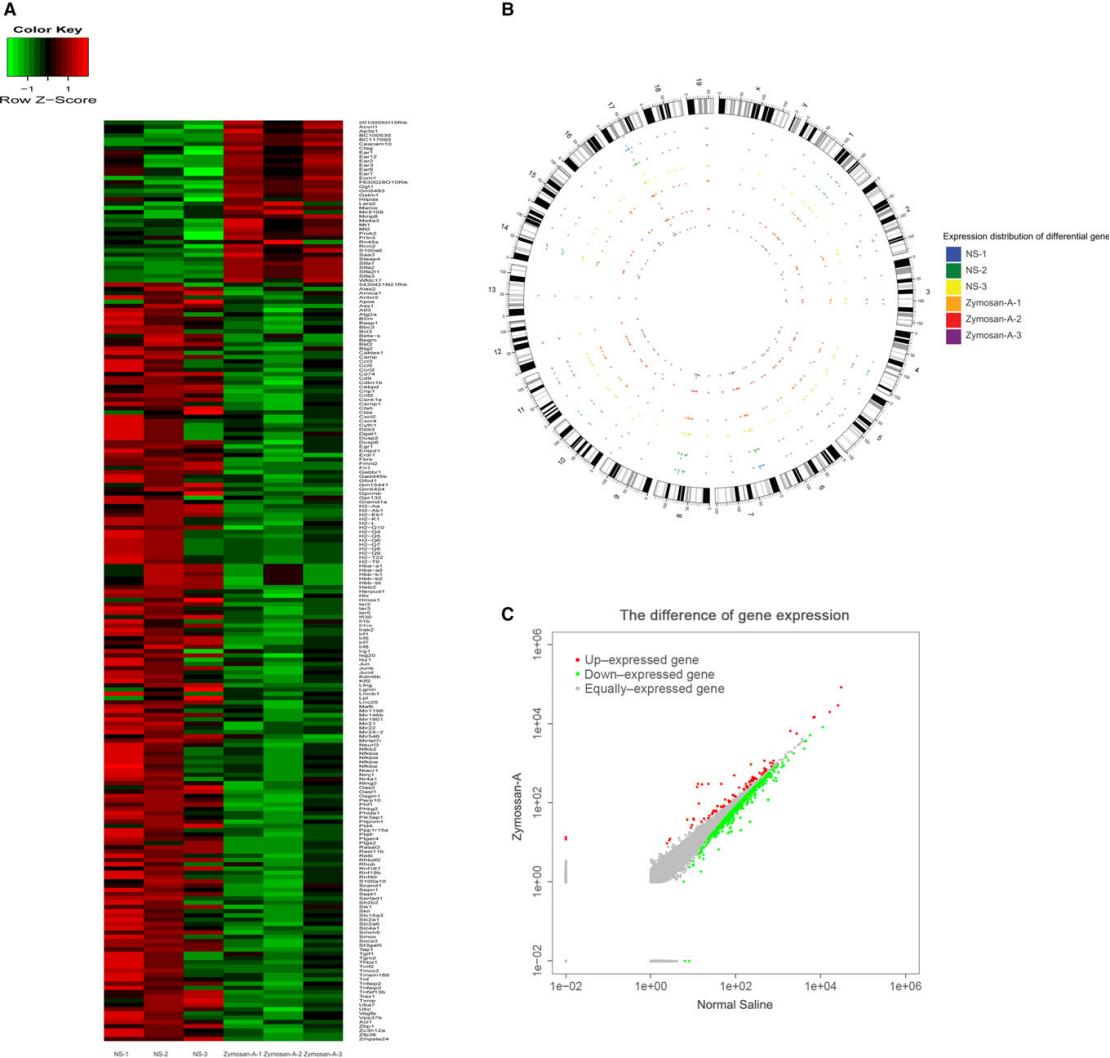
Identification of differentially expressed genes (DEGs) between IR + NS and IR + Zymosan‐A groups. (A) Heat map of DEGs (30 up‐regulated genes and 101 down‐regulated genes. Red: up‐regulation; Green: down‐regulation). (B) Expression distribution of differential gene. (C) The scatter plots of DEGs (Red: up‐expressed gene; Green: down‐expressed gene; Grey: equally expressed gene).

Using the RNA sequencing technology, total of 131 DEGs were identified ([log2FoldChange] >.8 and *p*‐value <.05), including 30 up‐regulated genes and 101 down‐regulated genes in the IR + Zymosan‐A groups, compared to IR + NS groups (Table 1). DEGs expression heat map was shown in Figure 4.

**Table 1:** One hundred and thirty‐one differentially expressed genes (DEGs) were identified between IR + NS and IR + Zymosan‐A groups

Up‐regulated gene		Down‐regulated gene						
Stfa2	Ecm1	Egr1	Herpud1	Ccrl2	Zc3h12a	Ier3	Irak2	Hbb‐b1
Stfa3	Mir5109	Atf3	Gramd1a	Xcl1	Socs3	Lfng	H2‐K1	Beta‐s
BC100530	F630028O10Rik	Ptafr	Lpl	Bpgm	Ier5	Cyth1	Cxcl2	Slc4a1
BC117090	Gstm1	Bcl3	Ptgs2	H2‐Q4	Tnf	Niacr1	Tnfsf13b	Mir21
2010005H15Rik	Ear1	Rasal3	Phf1	H2‐Q5	Erdr1	Txnip	Mir22	Hba‐a2
Stfa1	Mt1	Smox	Skil	Rasl11b	Nfkbia	H2‐Ab1	H2‐Eb1	Hba‐a1
Gm5483	Rn45s	Amica1	Cd74	Fmnl2	Mir24‐2	H2‐T22	Zfp36	Hbb‐b2
Stfa2 l1	Ear12	Neurl3	Nfkbid	Cables1	Relb	Nfkbiz	Nfkb2	Hbb‐bt
Saa3	Ear3	Ier2	Hmox1	Mir1901	Tnfaip3	H2‐T9	Ppp1r15a	Mirlet7i
Mt2	Ear7	5430421N21Rik	Klf2	Tmcc2	Fn1	Junb	Smim5	Gpnmb
Marco	Ear6	Bbc3	Jund	H2‐Q6	H2‐Q10	Phlda1	Gabbr1	Mir146b
Ggt1	Acvrl1	Irg1	H2‐Aa	H2‐Q8	Thbs1	Gm15441	Mir1198	
Prok2	Ceacam10	Rnf167	Tgif1	H2‐Q9	Nfkbie	Jun	Dusp2	
Lars2	Ctsg	Pik3ap1	Tgm2	H2‐Q7	Gadd45b	Zmpste24	Antxr2	
Steap4	Ear2	Sh2b2	Sertad1	Alas2	Ptger4	Basp1	Ninj1	

### 3.5 DEGs gene ontology analysis between IR + NS and IR + Zymosan‐A groups

**Figure 5:**
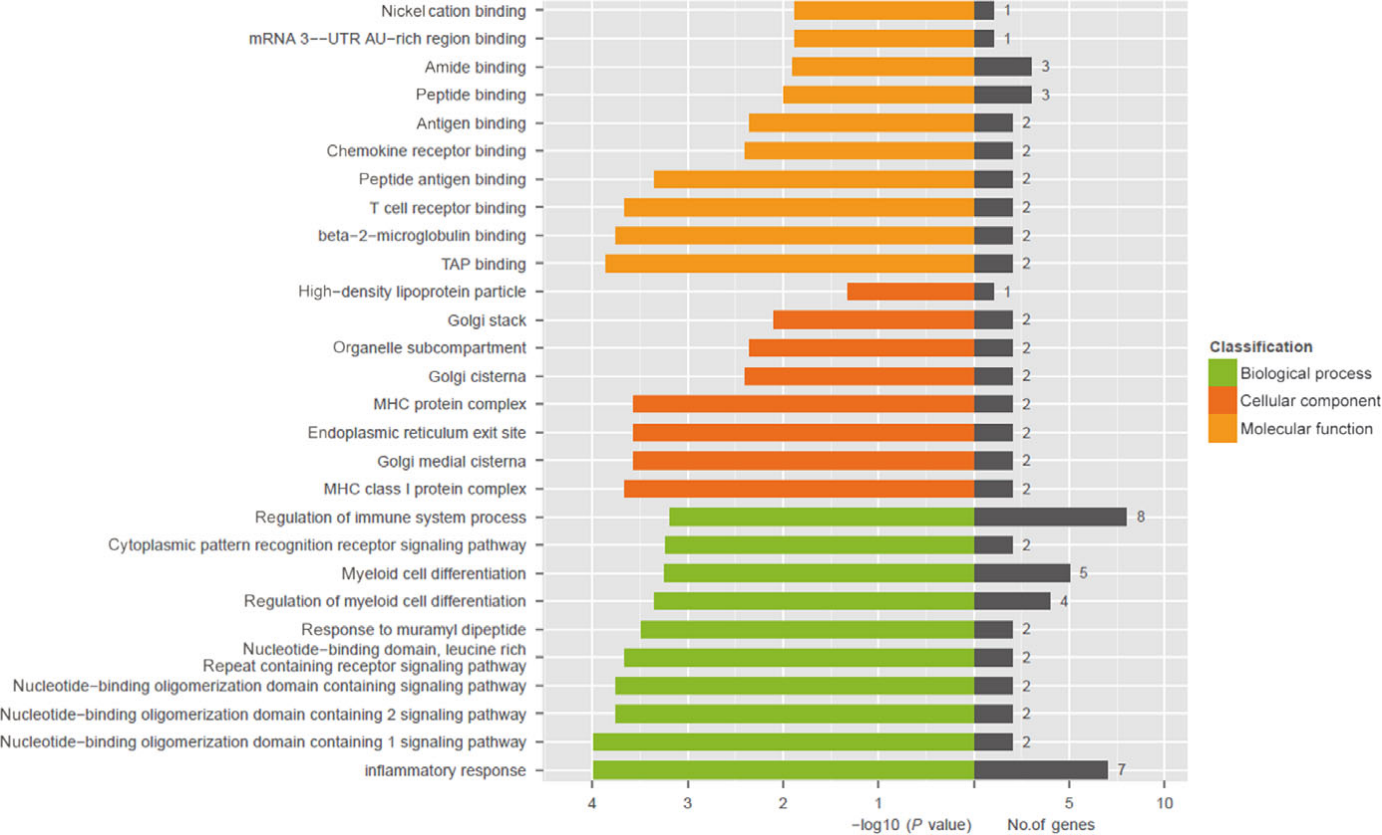
Gene ontology analysis and significant enriched GO terms of differentially expressed genes (DEGs) between IR + NS and IR + Zymosan‐A groups. GO analysis classified the DEGs into 3 groups (biological process group, molecular function group and cellular component group).

Gene ontology analysis was used to investigate changes in the patterns of genes between IR + NS and IR + Zymosan‐A groups. The significantly enriched GO analysis of DEGs was shown in Table 2 and Figure 5. The DEGs were classified into 3 functional groups: biological process group, cellular component group and molecular function group. The inflammatory response, nucleotide‐binding oligomerization domain containing 1 pathway and nucleotide‐binding oligomerization domain containing 2 pathways were significantly enriched in biological process group. The MHC class 1 protein complex, Golgi cisternae and endoplasmic reticulum exit site were significantly enriched in cellular component group. Within the molecular function group, the TAP binding, beta‐2‐microglobulin binding and peptide antigen binding were significantly enriched.

**Table 2:** The differentially expressed genes were classified into 3 functional groups: biological process group, cellular component group and molecular function group

	Term	Sample number	*P*‐value
GOBPID			
GO:0006954	Inflammatory response	7	.000101907
GO:0070427	Nucleotide‐binding oligomerization domain containing 1 signalling pathway	2	.000102889
GO:0070431	Nucleotide‐binding oligomerization domain containing 2 signalling pathway	2	.000175861
GO:0070423	Nucleotide‐binding oligomerization domain containing signalling pathway	2	.000175861
GO:0035872	Nucleotide‐binding domain, leucine rich repeat containing receptor signalling pathway	2	.000219501
GO:0032495	Response to muramyl dipeptide	2	.000320983
GO:0045637	Regulation of myeloid cell differentiation	4	.000441585
GO:0030099	Myeloid cell differentiation	5	.000561916
GO:0002753	Cytoplasmic pattern recognition receptor signalling pathway	2	.000580165
GO:0002682	Regulation of immune system process	8	.00065509
GOCCID			
GO:0042612	MHC class I protein complex	2	.000219501
GO:0005797	Golgi medial cisterna	2	.000267882
GO:0070971	Endoplasmic reticulum exit site	2	.000267882
GO:0042611	MHC protein complex	2	.000267882
GO:0031985	Golgi cisternae	2	.004006148
GO:0031984	Organelle subcompartment	2	.00438872
GO:0005795	Golgi stack	2	.008020845
GO:0034364	High‐density lipoprotein particle	1	.047761288
GOMFID			
GO:0046977	TAP binding	2	.000136983
GO:0030881	Beta‐2‐microglobulin binding	2	.000175861
GO:0042608	T cell receptor binding	2	.000219501
GO:0042605	Peptide antigen binding	2	.000441261
GO:0042379	Chemokine receptor binding	2	.004006148
GO:0003823	Antigen binding	2	.00438872
GO:0042277	Peptide binding	3	.009990679
GO:0033218	Amide binding	3	.012628348
GO:0035925	mRNA 3′‐UTR AU‐rich region binding	1	.013258393
GO:0016151	Nickel cation binding	1	.013258393

### 3.6 Signalling pathway enrichment analysis of DEGs between IR + NS and IR + Zymosan‐A groups

**Figure 6:**
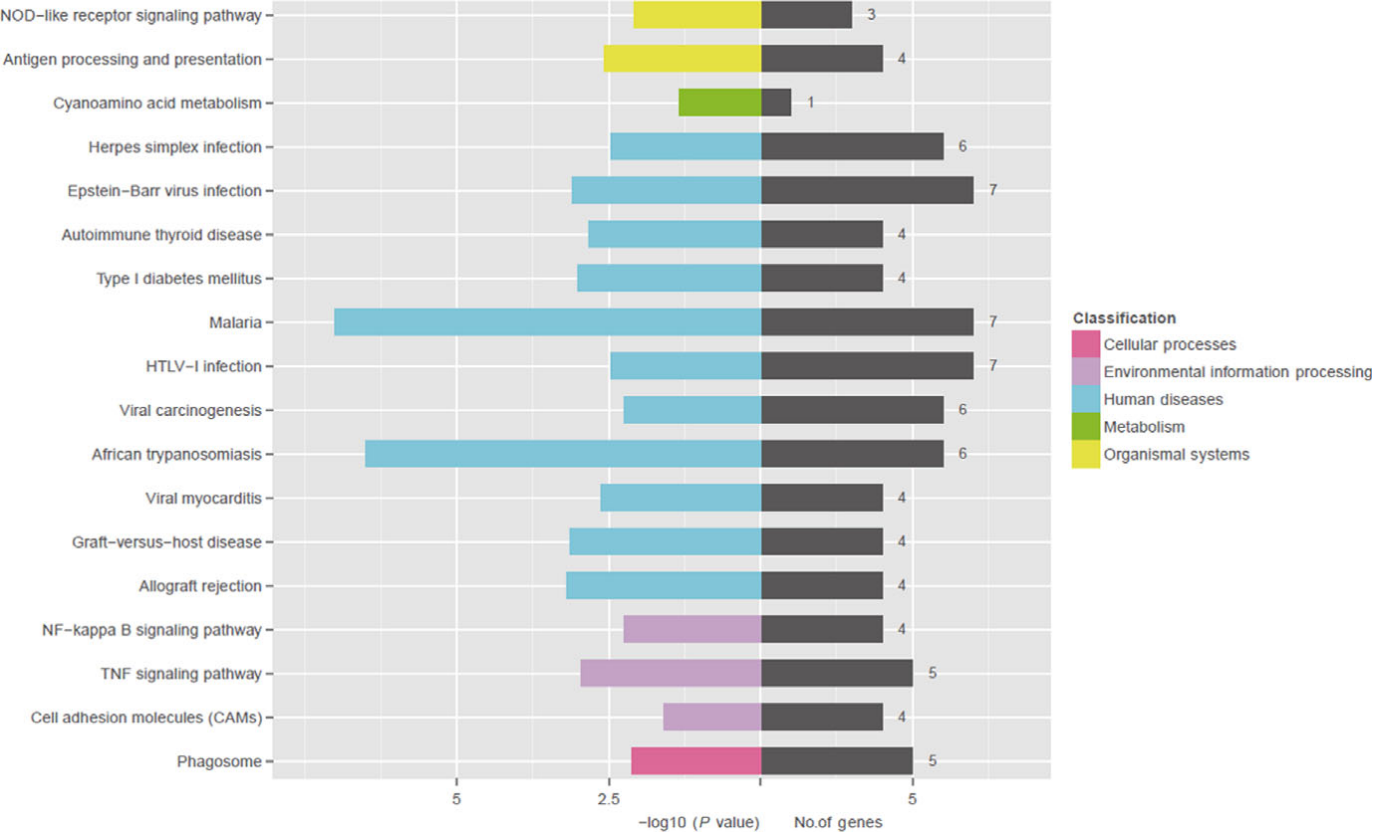
Significant enriched GO terms of differentially expressed genes (DEGs) between IR + NS and IR + Zymosan‐A groups. KEGG analysis classified the DEGs into 5 groups (Cellular processes, environmental information, human diseases, metabolism and organismal systems).

To further study the biological functions of the DEGs, KEGG analysis was used to evaluate the functions of the DEGs. The DEGs were mapped to 18 pathways in the KEGG database, as shown in Table 3. Moreover, the DEGs were classified into 5 classifications including cellular processes, environmental information, human diseases, metabolism and organismal systems (Figure 6). Within the environmental information group, TNF signalling pathway and NF‐kappa B signalling pathway were significantly enriched.

**Table 3:** Signalling pathway enrichment analysis of differentially expressed genes between IR + NS and IR + Zymosan‐A groups

Term	Id	Sample number	*P*‐value
Malaria	mmu05144	7	9.88E‐08
African trypanosomiasis	mmu05143	6	3.19E‐07
Allograft rejection	mmu05330	4	.000639
Graft‐versus‐host disease	mmu05332	4	.000727
Epstein‐Barr virus infection	mmu05169	7	.000801
Type I diabetes mellitus	mmu04940	4	.000983
TNF signalling pathway	mmu04668	5	.001113
Autoimmune thyroid disease	mmu05320	4	.001515
Viral myocarditis	mmu05416	4	.002323
Antigen processing and presentation	mmu04612	4	.002648
HTLV‐I infection	mmu05166	7	.003374
Herpes simplex infection	mmu05168	6	.003388
NF‐kappa B signalling pathway	mmu04064	4	.005635
Viral carcinogenesis	mmu05203	6	.005662
Phagosome	mmu04145	5	.007443
NOD‐like receptor signalling pathway	mmu04621	3	.008153
Cell adhesion molecules (CAMs)	mmu04514	4	.025283
Cyanoamino acid metabolism	mmu00460	1	.04628

## 4 DISCUSSION

Radiation‐induced death is a complex pathophysiological process.^6^, ^25^ The loss of BMC is the major reason of mice death after radiation. In previous study, we showed that the number of BMCs in mice treated with Zymosan‐A was higher than that in mice treated with NS.^19^ Using flow cytometry, we proved that Zymosan‐A inhibited apoptosis of BMCs induced by radiation, which contributed to the increased number of BMCs. Next, we detected the levels of GM‐CSF, G‐CSF, IL‐6 and IL‐12 in BMCs. Those cytokines play important roles in haematopoiesis.^26^, ^27^ For example, G‐CSF, which can stimulate the production of proteases that cleave many interactions including CXCR4/SDF‐1, has been used to induce HSC mobilization in current clinical practice.^28^ In addition, our unpublished experimental data revealed that Zymosan‐A also increased the number of LSK cells, which play critical roles in reconstitution of haematopoietic system after radiation.^29^, ^30^

Ionizing radiation can lead to DNA damage through direct and indirect effects, including double‐strand breaks(DSB), single‐strand breaks(SSB), base mutation and deletion.^31^ Among them, DSB is the most serious consequence of cell, which can directly cause cell death and induce a series of inflammatory responses.^32^, ^33^ In our previous study, we found that Zymosan‐A can protected AHH‐1 form radiation‐induced apoptosis.^19^ By detecting γ‐H2AX, the marker of DSB damage, we showed that the DSB damage can be significantly reduced in Zymosan‐A‐treated group. This date reminded us that the radioprotection of Zymosan‐A can be achieved by reducing DSB.

RNA sequencing technology is highly accurate, rapid and effective. Using the RNA sequencing, 131 DEGs were identified finally and most of them were down‐regulated. Moreover, the DEGs were classified into 3 functional groups using GO analysis. And, many DEGs were enriched in the GO term of biological process group, especially evolved in the regulation of immune system process and inflammatory response. Our previous study revealed that Zymosan‐A protected the haematopoietic system from radiation‐induced damage via Toll‐Like Receptor 2. Thus, combining this study and our previous study, we concluded that Zymosan‐A might exhibit radioprotective effect through regulating immune system process and inflammatory response, in which TLR2 showed a key role KEGG analysis also supported this view, and showed that NOD‐like receptor signalling pathway, TNF signalling pathway and NF‐kappa B signalling pathway were significantly enriched in the environmental information group. Consistent with the finding using KEGG analysis, TNF and NF‐kappa B signalling pathway has been demonstrated that it was associated with radioprotection in our previous work.^17^ Thus, this study revealed the gene network of protective effects of Zymosan‐A.

In conclusion, Zymosan‐A exhibited great protective effects against ionizing radiation. Zymosan‐A protected bone marrow cells from radiation‐induced apoptosis, up‐regulated IL‐6, IL‐12, G‐CSF and GM‐CSF in BMCs. In addition, Zymosan‐A also protected cells from radiation‐induced DNA damage in vitro. Moreover, Zymosan‐A treatment induced 131 DEGs which were related to immune system process and inflammatory responses. These results provided new knowledge regarding the radioprotective effect of Zymosan‐A.

## CONFLICT OF INTEREST

The authors have no potential conflict of interest to disclose.
